# DPHB, a diarylheptane from *Alpinia officinarum* Hance, ameliorates insulin resistance: A network pharmacology and *in vitro* study

**DOI:** 10.3389/fphar.2022.956812

**Published:** 2022-09-01

**Authors:** Xiangyi Li, Huan Wen, Yuxin Zhang, Aixia Liu, Xuguang Zhang, Minghai Fu, Yipeng Pan, Jian Xu, Junqing Zhang

**Affiliations:** ^1^ Key Laboratory of Tropical Translational Medicine of Ministry of Education, Hainan Provincial Key Laboratory for Research and Development of Tropical Herbs, Haikou Key Laboratory of Li Nationality Medicine, School of Pharmacy, Hainan Medical University, Haikou, China; ^2^ Department of Transplantation, The Second Affiliated Hospital of Hainan Medical University, Haikou, China

**Keywords:** network pharmacology, molecular docking, (4E)-7-(4-hydroxy-3-methoxyphenyl)-1-phenylhept-4-en-3-one, *Alpinia officinarum* Hance, insulin resistance

## Abstract

(4*E*)-7-(4-Hydroxy-3-methoxyphenyl)-1-phenylhept-4-en-3-one (DPHB) derived from *A. officinarum* Hance has been reported to exert anti-inflammatory and anti-insulin resistance (IR) effects. We explored the molecular mechanism of DPHB ameliorating IR through network pharmacological prediction and *in vitro* analysis. The PI3K/AKT and TNF signaling pathways are the core pathways for DPHB to exert anti-IR, and the key proteins of this pathway were confirmed by molecular docking. In the IR-3T3-L1 adipocyte model, DPHB significantly promoted glucose uptake and the glucose transporter type 4 (GLUT4) translocation. In addition, DPHB significantly improved lipid accumulation, triglyceride content, and the mRNA expression of key adipokines [such as *peroxisome proliferator-activated receptors-gamma* (PPARγ), *CCAAT enhancer*-*binding protein alpha* (C/EBPα), and *sterol regulatory element-binding protein-1* (SREBP-1)]. DPHB inhibited the protein expression of tumor necrosis factor-α (TNF-α), interleukin-6 (IL-6), and phosphorylated nuclear factor-κB (NF-kB), as well as promoted the expression of phosphatidylinositol 3-kinase (PI3K), protein kinase B (AKT), phosphorylated PI3K, and phosphorylated AKT. More interestingly, validation of the PI3K inhibitor LY294002 revealed that these changes were dependent on the activation of PI3K. Our cumulative findings thereby validate the potential of DPHB to alleviate and treat IR and the related diseases by regulating the PI3K/AKT and TNF-α signaling pathways.

## 1 Introduction

Insulin resistance (IR), which leads to type 2 diabetes mellitus and several metabolic disorders, is characterized by the decreased ability of insulin-target tissues (such as skeletal muscle, liver, and adipose tissue), to respond to the stimulation by insulin (Daryabor et al., 2020). IR is usually accompanied by hyperglycemia, dyslipidemia, endothelial dysfunction, and elevated levels of inflammatory markers (Kitada et al., 2019). Overcoming insulin resistance might prevent many such metabolic diseases. IR is characterized by impaired insulin signaling leading to the failure of GLUT4 in target tissues consequently hindering glucose and lipid homeostasis (James et al., 2021). IR is also associated with obesity and chronic, low-grade inflammation. Adipokines (such as PPARγ, C/EBPα, and SREBP-1) are involved in the proliferation, differentiation, and expansion of adipocytes. Over-expanded adipocytes could lead to cell hypoxia and stimulate adipose tissue to secrete inflammatory factors (such as TNF-α and IL-6). The occurrence of inflammation and obesity reduces the body’s insulin sensitivity and aggravates IR (Yazıcı and Sezer, 2017; Kojta et al., 2020). The long-term use of clinical drugs like metformin and thiazolidinediones, which are used to treat IR, may cause severe side effects such as drug resistance or heart failure (Chaudhury et al., 2017). Therefore, exploring a therapeutic drug with lower side effects, better efficacy, and higher safety is of great significance for the treatment of IR.

Traditional Chinese medicine (TCM) and natural products have several advantages like reduced toxicity, lower side effects, and long-lasting effects; hence, their medicinal properties are being explored widely. Identification of suitable alternatives from TCM and natural products for the treatment of IR will significantly improve the management of IR and related metabolic disorders. *A. officinarum* Hance (*A. officinarum*), a dry rhizome from the Zingiberaceae family, is widely used in TCM and Ayurvedic medicine. It is also widely used in food ingredients and flavorings (Dong et al., 2015; Huang et al., 2016). Modern pharmacological studies have shown that *A. officinarum* has various biological activities, such as antioxidant, anti-gastric ulcer, antibacterial, anti-diabetic, anti-hypertensive, and diuretic activities (Abubakar et al., 2018; Javaid et al., 2021). Moreover, *A. officinarum* is often used to treat diabetes, stomach pain, swelling, and colds in China and Europe (Basri et al., 2017). Pharmacodynamic studies have proved that diarylheptane, which has several biological activities, is one of the main active ingredients in *A. officinarum* rhizome (Lin et al., 2020; Liu et al., 2020). Our previous studies also demonstrated that DPHC from *A. officinarum* could decrease IR by reducing the blood glucose level in db/db mice and increasing the glucose uptake in high-glucose-treated HepG2 cells (Zhang et al., 2022). Furthermore, we found that (4*E*)-7-(4-hydroxy-3-methoxyphenyl)-1-phenylhept-4-en-3-one (DPHB), another diarylheptane compound from *A. officinarum*, may also help in improving IR. In our preliminary experiment, DPHB treatment significantly increased glucose uptake and maintained near basal glucose levels in IR-3T3-L1 adipocytes. However, the underlying molecular mechanism or the mode of action of DPHB is unknown.

Network pharmacology, in combination with the system biology theory, utilizes high-throughput omics data analysis, computer simulation, network database retrieval, and other technologies to construct drug–target–disease interaction networks as well as to systematically clarify the potential targets and pharmacological effects of traditional Chinese medicine (TCM) components to evaluate the role of TCM in diseases (Nogales et al., 2022). Therefore, an increasing number of studies have applied network pharmacology to clarify the pharmacodynamic basis and the molecular mechanism of TCM/drugs in disease treatment (Luo et al., 2020; Wu et al., 2020; Cai et al., 2021).

In this study, a method based on network pharmacology-integrated molecular docking was used to identify the potential targets and pharmacological mechanisms of DPHB. An *in vitro* study conducted on the IR-3T3-L1 adipocyte model confirmed the relieving effects and molecular mechanisms of DPHB on IR. These findings provide a reliable reference for the therapeutic research and clinical application of DPHB in IR.

## 2 Materials and methods

### 2.1 Drug extraction and isolation

**FIGURE 1:**
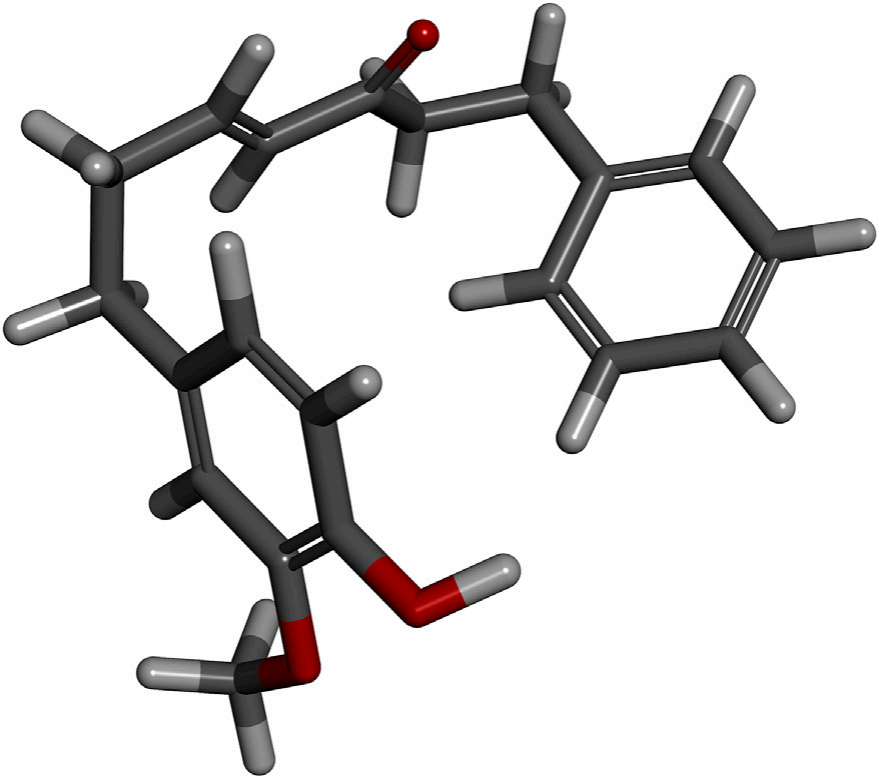
Three-dimensional spatial structure of DPHB. Red sticks: oxygen atoms; white sticks: hydrogen atoms; and gray sticks: carbon atoms.

The *A. officinarum* Hance powder (42 kg) was extracted with 85% ethanol (material:liquid ratio = 1:10) by the heating reflux method thrice, 2 h each time. The solvents were combined and concentrated to 20 L to obtain the crude extract, which was then extracted with ethyl acetate (EtOAc) to obtain the ethyl acetate extract. The ethyl acetate extract obtained was chromatographed on a silica gel (200–300 mesh) column and eluted with petroleum ether/EtOAc (30:1–15:1, v/v). When the column was eluted at a ratio of 15:1, the most bioactive compound (4*E*)-7-(4-hydroxy-3-methoxyphenyl)-1-phenylhept-4-en-3-one (DPHB) was obtained, which was based on spectroscopic analysis (13C NMR and 1H NMR) to confirm the structure. High-performance liquid chromatography (HPLC) determined the purity of DPHB as being >98%. The chemical structure of DPHB is shown in Figure 1.

### 2.2 Network pharmacology

#### 2.2.1 Potential target proteins of DPHB and IR

After drawing the chemical structure of DPHB in SwissTargetPrediction (http://swisstargetprediction.ch/; access time: 2022-03-07) (Daina et al., 2019), the potential target of DPHB for *Homo sapiens* was predicted, and the target with probability >0 was selected. The potential targets of DPHB were supplemented by submitting the 2D chemical structure files of DPHB in PharmMapper (http://www.lilab-ecust.cn/pharmmapper/; access time: 2022-03-11) (Wang et al., 2017) and selecting human protein targets alone. Then, the targets in these databases were merged and used as the related target of DPHB after removing the redundancy.

In addition, “insulin resistance” was used as the keyword to screen out the related targets of IR from the web databases, including the GeneCards database (https://www.genecards.org/; access time: 2022-03-07), PharmGKB database (https://www.pharmgkb.org/; access time: 2022-03-07), Therapeutic Target Database (TTD, http://db.idrblab.net/ttd/; access time: 2022-03-07), DrugBank database (https://www.drugbank.ca/; access time: 2022-03-07), and Online Mendelian Inheritance in Man database (OMIM, https://www.omim.org/; access time: 2022-03-07) (Stelzer et al., 2016; Wishart et al., 2018; Amberger et al., 2019; Whirl-Carrillo et al., 2021; Zhou et al., 2022). Moreover, for the GeneCards databases, targets with a “relevance score” >10 were preserved for further investigations. All the targets were standardized into official gene symbols concerning the UniProt database (https://www.uniprot.org/; access time: 2022-03-09) (UniProt Consortium. 2021). Then, the data obtained were combined to remove all duplicate entries as relevant targets for IR. Finally, the results obtained were visualized using the EVenn network tool (http://www.ehbio.com/test/venn/#/) (Chen et al., 2021).

In order to acquire the DPHB common target in the amelioration of IR, the EVenn network tool was used to construct a Venn diagram of the DPHB target genes and the IR target genes, and the overlaps were considered the potential targets of DPHB to improve IR.

#### 2.2.2 Protein–protein interaction network

The protein–protein interaction (PPI) data for the intersection targets of DPHB and IR were obtained from the STRING database (https://string-db.org) (Szklarczyk et al., 2021). The PPI data were collected and imported into Cytoscape 3.8.2 software to create the PPI network diagram. Then, the core targets of the network were screened by the degree (D), betweenness centrality (BC), and closeness centrality (CC) using the CytoNCA analysis (Lotia et al., 2013; Otasek et al., 2019).

#### 2.2.3 Gene ontology (GO) and kyotoencyclopedia of genes and genomes (KEGG) pathway enrichment analyses

GO function analysis and KEGG pathway enrichment analysis were performed on the potential targets by using the Metascape database (http://metascape.org/gp/index.html#/) (Zhou et al., 2019). Considering *p* < 0.05 as the screening condition, the top 20 signaling pathways and the top 10 GO terms were selected. Moreover, the GO terms were divided into three aspects, namely, the cellular component (CC), molecular function (MF), and biological process (BP).

#### 2.2.4 Construction of a network of “component–target–pathway”

The “component–target–pathway” (C-T-P) network of DPHB to improve IR was constructed using Cytoscape 3.8.2 software. The nodes of the network were mainly composed of DPHB, potential targets, the top 20 signaling pathways, and their targets screened by the KEGG pathway enrichment analysis. Moreover, the core target was further examined by the degree value of the node.

#### 2.2.5 Molecular docking

The structures of DPHB were painted in Discovery Studio 2019 (Kemmish et al., 2017). The crystal structures of AKT1 (PDB ID: 6HHI), PIK3CA (PDB ID: 6AUD), TNF (PDB ID: 6X83), IL-6 (PDB ID: 5FUC), and RELA (NF-kB p65; PDB ID: 6QHL) were downloaded from the RCSB database (http://www.RCSB.org) (Burley et al., 2021). The proteins were added to Discovery Studio 2019 to remove water molecules and ligands from the environment and to define their binding sites. Then, DPHB and proteins were analyzed by Discovery Studio 2019, and their docking site and the binding capacity of the ligand and protein were recorded.

### 2.3 *In vitro* study

#### 2.3.1 Cell culture

The 3T3-L1 murine fibroblastic cell line was obtained from Shanghai Biological Technology Co., Ltd. enzyme res (Shanghai, China) and cultured in high-glucose Dulbecco’s modified essential medium (DMEM, Gibco, United States) supplemented with 10% calf serum (CS; Gibco) and 1% penicillin–streptomycin (Biosharp, Anhui, China) in a cell incubator maintained at 37°C and 5% CO_2_. To obtain mature adipocytes, a previously described method was used, albeit with some modifications (Wang et al., 2020). Briefly, 3T3-L1 was seeded at 5 × 10^4^ cells/cm^2^ concentration in a multiwell plate and incubated for 3 days for confluence. Then, after 4 days of culture in the DMEM supplemented with 10% fetal bovine serum (FBS, Gibco), 0.5 mM 3-isobutyl-1-methylxanthine (IBMX), 1 µM dexamethasone (Dex), and 10 μg/ml insulin, the medium was changed to a DMEM supplemented with 10% FBS and 10 μg/ml insulin for 4 days, and finally, the cells were cultured in DMEM supplemented with 10% FBS until 90% of the cells exhibited an adipocyte phenotype.

In order to examine the effect of DPHB on IR, an IR model was established with 1 µM Dex cultured for 96 h (Yuan et al., 2019), and 1–30 µM DPHB or 20 µM rosiglinone (Rosi) was administered to intervene 16 h before the end of the experiment. Then, the experimental cells were assigned to the control group (DMEM), the model group (DMEM + 1 µM Dex), the Rosi group (DMEM + 1 µM Dex + 20 µM Rosi), the 1-µM DPHB group (DMEM + 1 µM Dex + 1 µM DPHB), the 10-µM DPHB group (DMEM + 1 µM Dex + 10 µM DPHB), and the 30-µM DPHB group (DMEM + 1 µM Dex + 30 µM DPHB). In addition, to investigate whether the anti-IR effect of DPHB is dependent on PI3K, the PI3K inhibitor LY294002 (LY, 10 µM) was added as an additional experimental group with or without DPHB (30 µM) and subjected to Western blotting and quantitative polymerase chain reaction (qPCR) experiments.

#### 2.3.2 Cell viability assay

Cell viability was tested using the cell counting kit-8 assay (CCK-8 assay; Beyotime, Shanghai, China). Then, 3T3-L1 preadipocytes were seeded at the concentration of 1 × 104 cells/well in a 96-well plate and cultured in a DMEM supplemented with 10% CS. After 24 h of cell adhesion, the cells were treated with different concentrations of DPHB (1–100 μM) for 16 h. Then, 10 μl of the CCK8 solution was added to each well at 37°C for 1 h. The absorbance was measured at 450 nm using the SpectraMax Plus Automatic Plate Reader (Molecular Devices, Sunnyvale, CA, United States).

#### 2.3.3 Glucose uptake assay

Glucose uptake assay was performed using the fluorescence probe (2-NBDG, Invitrogen, United States), and 3T3-L1 preadipocytes were cultured at the concentration of 5 × 104 cells/well in a six-well plate until differentiation. After induction of IR and pretreatment of the compounds, the differentiated 3T3-L1 adipocytes were stimulated with low-glucose DMEM containing or without 100 nmol/L insulin for 30 min, followed by incubation with 25 mM 2-NBDG per well for 30 min. Next, the cells were washed with cold PBS and collected, followed by fluorescence intensity analysis using a flow cytometer (FACSCalibur, Agilent NovoCyte Penteon, United States) at an emission wavelength of 530 nm and an excitation wavelength of 485 nm.

#### 2.3.4 Oil red O staining

The effect of DPHB on adipogenesis was evaluated by oil red O staining (Solarbio, Beijing, China). The 3T3-L1 preadipocytes were cultured in a 12-well plate until differentiation. After induction of IR and pretreatment of the compounds, the differentiated 3T3-L1 adipocytes were fixed with 10% formaldehyde for 15 min, followed by incubation with the filtered oil red O working solution (oil red O staining A: oil red O staining B = 3:2) for 30 min, washed with 60% isopropyl alcohol until colorless, and imaged using the AxioVertA1 inverted microscope (Zeiss, Oberkochen, Germany). To quantitatively analyze oil-red O staining, isopropanol was added to the stained cells to dissolve the dye completely, after which the staining was quantified using a microporous plate spectrophotometer at the detection wavelength of 510 nm.

#### 2.3.5 Triglyceride assay

To evaluate the intracellular triglyceride (TG) content, 3T3-L1 preadipocytes were cultured in 25-cm^2^ cell culture flasks, as described for the differentiation process. After induction of IR and pretreatment of the compounds, the cells were harvested with ice-cold PBS. After adding 1 ml of the TG extraction reagent (n-heptane:isopropanol = 1:1), the cells were disrupted using a cell sonicator (Q700, Qsonica, United States), and the supernatant was collected after centrifugation at 4°C. The total TG content in the supernatant was determined using a TG assay kit (Enzyme-linked Biotechnology Co., Ltd., Shanghai, China).

#### 2.3.6 Adiponectin secretion assay

3T3-L1 preadipocytes were cultured in a six-well plate until differentiation. After induction of IR and pretreatment of the compounds, the cell supernatant was collected, and the amount of adiponectin secreted by the adipocytes was determined using a mouse adiponectin enzyme-linked immunosorbent assay (ELISA) kit (Enzyme-linked Biotechnology Co., Ltd.).

#### 2.3.7 RNA extraction and qPCR analysis

The expression levels of *PPARγ*, *C/EBPα*, *SREBP-1*, *GLUT4*, *AKT*, and *PI3K* were detected by qPCR, and the extraction of total RNA from the 3T3-L1 adipocytes was performed by the application of Eastep^®^ Super Total RNA Extraction Kit (Shanghai Promega Biological Products Co., Ltd., China) as per the protocol defined by the manufacturer. The NanoPhotometer-N50 ultra-microspectrophotometer (Implen, Germany) was used to determine the quantity of the extracted RNA. The PCR amplification was performed under the following conditions: an initial activation of the hot-start DNA polymerase at 95°C for 5 min, followed by 40 cycles of the second step (i.e., 95°C for 10 s, 60°C for 20 s, and 72°C for 20 s). Next, by the melting curve stage, the amplification primers were obtained. The relative expression of each gene was analyzed by the 2^−ΔΔCt^ method, and all genes were normalized to their GAPDH levels. The qPCR primer sequences used in this study are shown in Table 1.

**TABLE 1:** qPCR primer sequences.

	Primer sequence-F (5′–3′)	Primer sequence-R (5′–3′)
AKT	TGC​ACA​AAC​GAG​GGG​AAT​ATA​T	CGT​TCC​TTG​TAG​CCA​ATA​AAG​G
PPARγ	GGA​AGA​CCA​CTC​GCA​TTC​CTT	GTA​ATC​AGC​AAC​CAT​TGG​GTC​A
C/EBPα	GCG​GGA​ACG​CAA​CAA​CAT​C	GTC​ACT​GGT​CAA​CTC​CAG​CAC
SREBP-1	TGA​CCC​GGC​TAT​TCC​GTG​A	CTG​GGC​TGA​GCA​ATA​CAG​TTC
GLUT4	GGT​TCC​TTG​GGT​TGT​GGC​AG	CTG​GAA​ACC​CGA​CGG​CAT​CTT​G
PI3K	CGA​GAG​TGT​CGT​CAC​AGT​GTC	TGT​TCG​CTT​CCA​CAA​ACA​CAG

#### 2.3.8 Western blotting

Total proteins from 3T3-L1 adipocytes were isolated by radioimmunoprecipitation assay (RIPA) lysis buffer (Beyotime, Haimen, China) containing phosphatase inhibitor (Beyotime, Haimen) and protease inhibitor cocktail (Biosharp). The membrane protein was extracted by using a membrane and cytosol protein extraction kit (Beyotime, Haimen). The protein concentration was determined by the BCA protein assay kit (Beyotime, Haimen). The proteins were separated by 8% or 12% sodium dodecyl sulfate-polyacrylamide gel and then transferred onto polyvinylidene difluoride membranes. The membranes were blocked with 5% nonfat dry milk for 1 h and incubated with different primary antibodies: NF-kB p65, *p*-NF-kB p65 (Ser536), IL-6, TNF-α (Affinity, Cambridge, United Kingdom), PI3K, *p*-PI3K (Tyr458/Tyr199), AKT, *p*-AKT (Ser473) (Cell Signaling Technology, Beverly, MA, United States), and GLUT4 (Proteintech, Chicago, IL, United States) at 4°C overnight. After washing with Tris-buffered saline containing Tween 20 (TBST), the membrane was incubated with the specific secondary antibody for 1 h at room temperature with shaking. Finally, the membrane surface was uniformly coated with the ECL chemiluminescent substrate reagent (Biosharp) and then exposed to the Image Lab analysis system (Bio-Rad, ChemiDocXRS+, United States). The protein expression was normalized to the tubulin level.

### 2.4 Statistical analysis

All experiments were repeated at least thrice. All data were analyzed using GraphPad Prism software (GraphPad software, CA, United States) and expressed as the mean ± standard deviation (SD). The nonpaired *t*-test was employed to compare differences and analyze the two groups (^#^ *p* < 0.05, ^##^ *p* < 0.01, and ^###^ *p* < 0.001, when compared with the control group; *p < 0.05, **p < 0.01, and ***p < 0.001, when compared with the model group; and ^+^ *p* < 0.05, ^++^ *p* < 0.01, and ^+++^ *p* < 0.001, when compared with the 30-μM DPHB group).

## 3 Results

### 3.1 Network pharmacology prediction

#### 3.1.1 Identification of the potential targets of DPHB that improve insulin resistance

**FIGURE 2:**
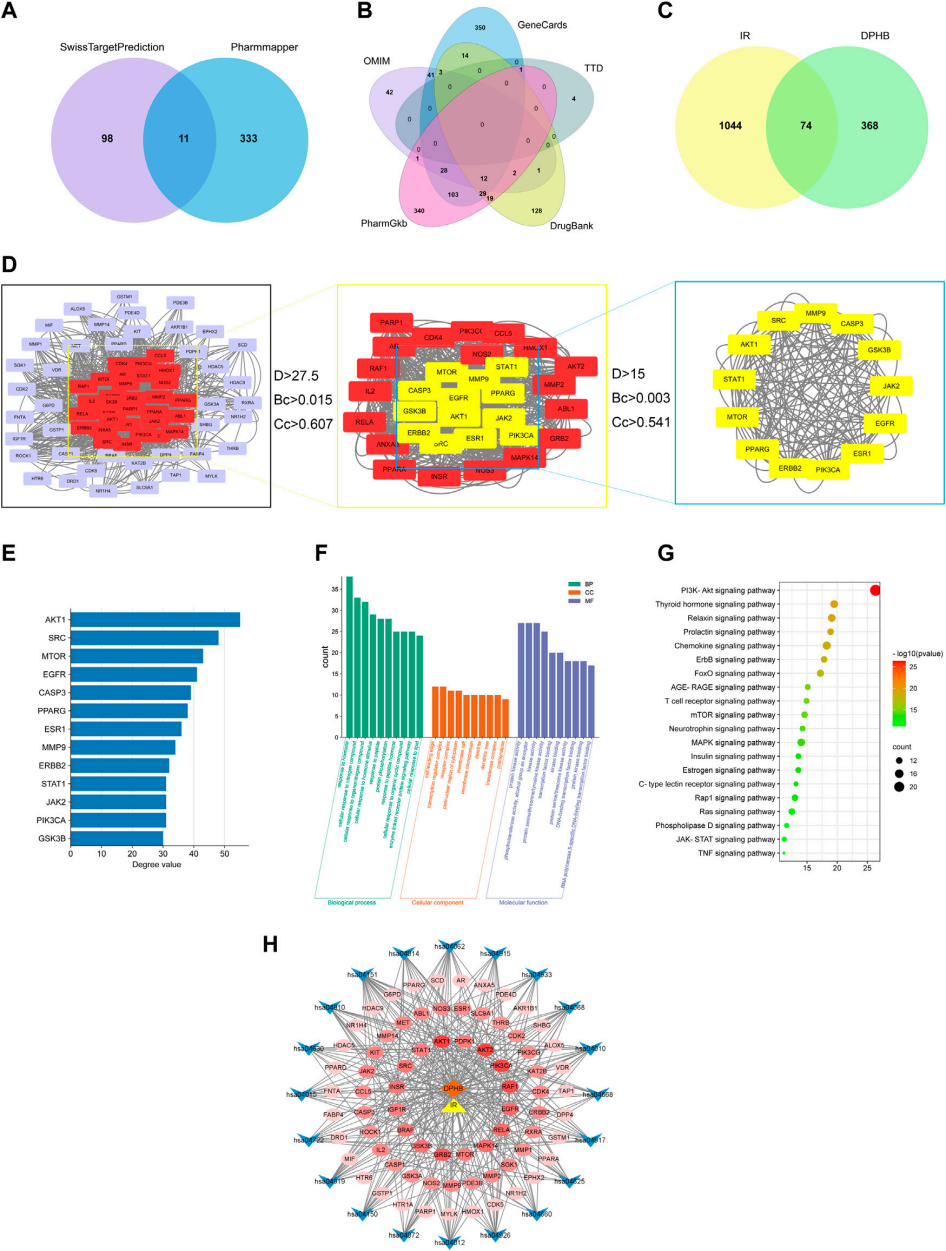
Network pharmacology analysis of DPHB on IR. **(A)** Venn diagram of the DPHB-related targets; **(B)** Venn diagram of the IR-related targets; **(C)** Venn diagram of the IR- and DPHB-related targets; **(D)** PPI network of the potential therapeutic targets; **(E)** degree value of 13 key nodes; **(F)** GO enrichment analysis of the targets of DPHB; **(G)** analysis of KEGG enrichment in 20 pathways as the targets of DPHB; **(H)** “component–target–pathway” of DPHB ameliorating IR. Blue nodes: the KEGG pathway of the KEGG-term identifier; red nodes: overlapping genes between DPHB and IR; the redder the color of the node, the higher the degree value of the node; orange node: DPHB; and yellow node: IR.

A total of 453 DPHB-related targets were screened through the SwissTargetPrediction and PharmMapper databases, and the number of targets was 109 and 344, respectively. After removing the duplicates, 442 targets were identified as DPHB-related targets (Figure 2A). For potential therapeutic targets of IR, 536, 582, 5, 208, and 130 targets were screened through the databases of PharmGKB, GeneCards, TTD, DrugBank, and OMIM, respectively. After removing the duplicates, 1,118 targets were considered potential therapeutic targets for IR (Figure 2B). Subsequently, the intersection of the targets of DPHB and IR was obtained, and a total of 74 targets were obtained, which can be regarded as DPHB-potential targets for improving the IR (Figure 2C).

#### 3.1.2 Protein–protein interaction network

After predicting reliable interaction information for 74 potential targets from the STRING database, a PPI network was constructed and analyzed by Cytoscape 3.8.2 software. After hiding the unconnected nodes, a PPI network consisting of 73 nodes and 1,266 edges was obtained; the larger the node, the more biological functions the node had in the PPI network (Figure 2D). Therefore, according to the results of degree, betweenness centrality, and closeness centrality, a total of 13 key nodes were further screened out, which were regarded as the core targets for DPHB against IR. In addition, the size of the degree value indicated the size of the node. After sorting the filtered nodes (Figure 2E), it can be found that the degree value of AKT1 was the largest.

#### 3.1.3 GO and KEGG pathway enrichment analyses

To explore the functions of 74 target genes shared by DPHB and IR, GO enrichment analysis was performed, which gave 1,741 GO items, including 1,138 BP, 44 CC, and 95 MF terms. Then, considering *p* < 0.05 as the screening criterion, the top 10 BPs indicated that the common genes of DPHB and IR were mainly involved in hormone response, lipid response, organic nitrogen compound response, and protein phosphorylation response (Figure 2F). The enrichment analysis of CC mainly involved the cell-leading edge, the perinuclear region of the cytoplasm, and the membrane raft. Meanwhile, the GO terms enriched for MF mainly included protein kinase binding, transcription factor binding, DNA-binding transcription factor binding, and protein kinase activity. Moreover, we identified 86 pathways through the KEGG pathway enrichment analysis. Considering the enrichment value as the reference basis, we determined the top 20 pathways as the core drug pathways (Figure 2G). The relative enrichment analysis revealed the following pathways: the PI3K-Akt signaling pathway, chemokine signaling pathway, MAPK signaling pathway, AGE-RAGE signaling pathway, TNF signaling pathway, and various hormone transduction signaling pathways.

#### 3.1.4 Construction of a network of “component–target–pathway”

The network diagram of the “component–target–pathway” reflects the interaction of DPHB and its potential targets with the core pathway (Figure 2H). As shown in this figure, the potential targets were closely linked to the core pathway, implying that DPHB improves IR through multiple targets and multiple pathways. Furthermore, according to the shades of the red nodes (targets), AKT1, AKT2, PI3KCA, and RELA were closely related to the core pathway, which may be the main core target of DPHB.

#### 3.1.5 Molecular docking

The aforementioned network pharmacology results revealed that AKT1, PI3KCA, and RELA were involved in multiple core pathways, and that RELA is the key target of TNF signaling. Meanwhile, phosphorylated NF-kB P65 upregulates the expression of TNF and IL-6, thereby reducing insulin sensitivity and downregulating phosphorylated AKT. Therefore, we believe that DPHB may improve IR through PI3K/AKT and TNF signaling pathways. In addition, molecular docking was performed on the key proteins (AKT1, PIK3CA, TNF, IL-6, and RELA) of the PI3K/AKT and TNF signaling pathways. The lower the binding energy, the higher the affinity between the receptor and the ligand, and the higher the possibility of their interaction. When the binding energy is < 0, the ligand and the receptor can bind freely. As shown in Table 2, the docking binding energies of DPHB with AKT1, PIK3CA, TNF, IL-6, and RELA were <0, indicating that DPHB has a good binding affinity with AKT1, PIK3CA, TNF, IL-6, and RELA receptor proteins, thereby yielding a spontaneous and stable binding. The interaction between the ligand and the receptor mainly included carbon–hydrogen bond, conventional hydrogen bond, and van der Waals, among others, wherein the conventional hydrogen bond acted as the main factor for the formation of protein–drug complexes.

**TABLE 2:** Docker energy of DPHB.

	CDocker energy	CDocker interaction energy
AKT1	−33.737	−42.3252
PIK3CA	−26.7127	−37.8019
TNF	−33.3634	−43.7091
IL-6	−28.1105	−42.5766
RELA	−27.7644	−37.1662

**FIGURE 3:**
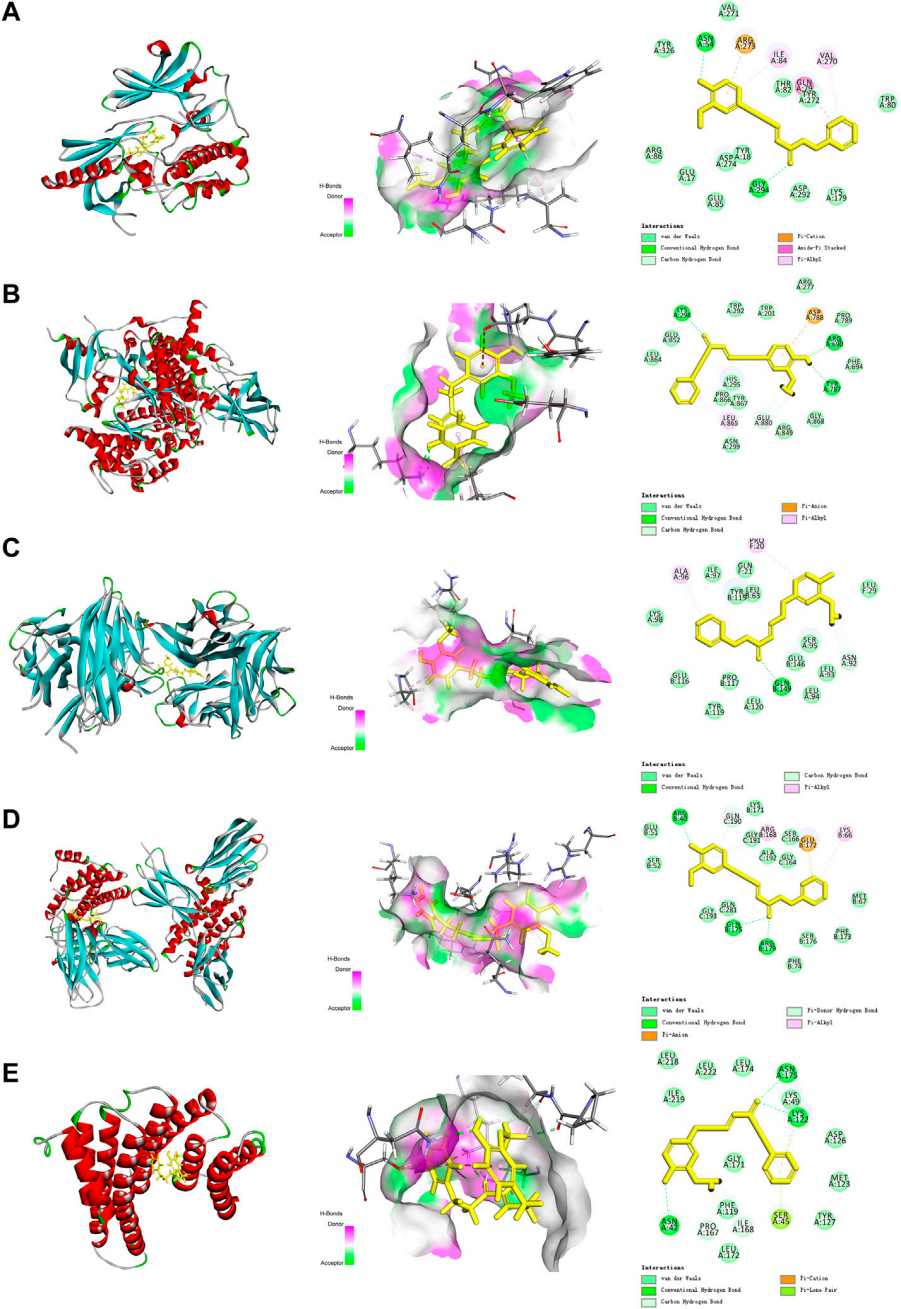
Molecular docking diagram of each target with DPHB. **(A)** AKT1; **(B)** PIK3CA; **(C)** TNF; **(D)** IL-6; and **(E)** RELA.

**FIGURE 4:**
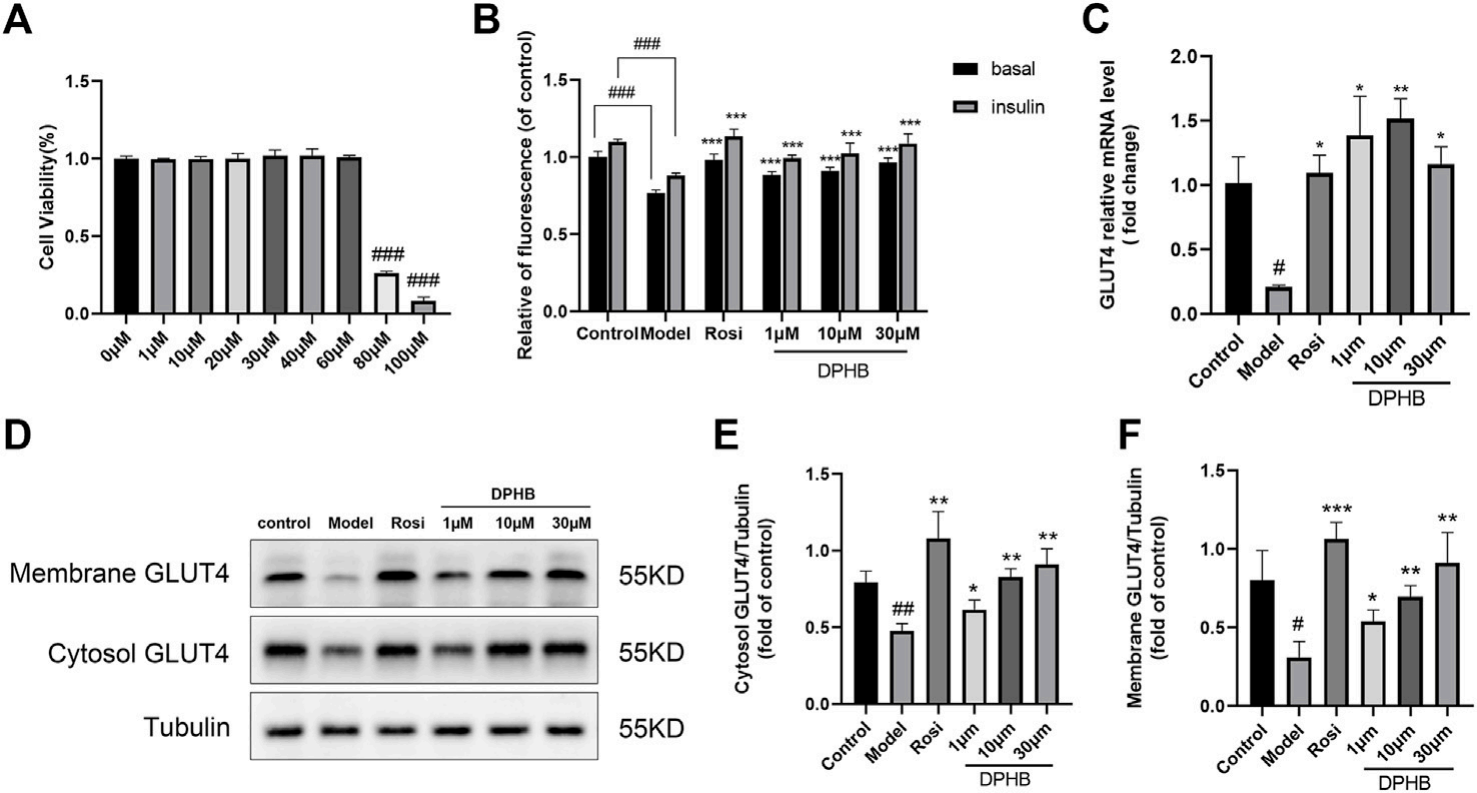
Effects of DPHB on glucose metabolism in IR-3T3-L1 adipocytes. **(A)** Effect of different concentrations of DPHB on the viability of 3T3-L1 adipocytes for 16 h; **(B)** quantifying fluorescence determined the extent of glucose uptake; **(C)** mRNA expression of GLUT4; **(D)** protein expression of GLUT4; **(E)** quantification of cytosol GLUT4; **(F)** quantification of membrane GLUT4. All values are expressed as the means ± SD, (*n* ≥ 3). *p < 0.05, **p < 0.01, and ***p < 0.001, when compared with the model control group; and ^#^ *p* < 0.05, ^##^ *p* < 0.01, and ^###^ *p* < 0.001, when compared with the control group.

As shown in Figure 4, DPHB formed three hydrogen bonds with LYS298, ARG690, and TYR787 of AKT (Figure 3A), two hydrogen bonds with ASN54 and GLY294 of PI3K (Figure 3B), three hydrogen bonds with ARG40, GLN175, and ARG179 of IL-6 (Figure 3D), and three hydrogen bonds with ASN175, LYS122, and ASN42 of RELA (Figure 3E). Moreover, DPHB formed a hydrogen bond with GLN149 of TNF and two pi-sulfur bonds with ALA96 and PRO20 (Figure 3C). This result further signified the spatial conformational relationship of DPHB binding to the aforementioned proteins, thereby validating the results of network pharmacology.

### 3.2 *In vitro* study

#### 3.2.1 Effect of DPHB on 3T3-L1 preadipocyte viability

In order to determine the toxicity of DPHB to 3T3-L1 preadipocytes and the administration concentration in the subsequent experiments, the CCK8 assay was performed to detect cell viability (Figure 4A). The results revealed that the concentration of 1–60 μM DPHB did not have any obvious effect on the viability of 3T3-L1 preadipocytes for 16 h. After the administration of 100 and 80 μM DPHB, cell viability was reduced to that of the 0-μM DPHB group (*p* < 0.001). Therefore, DPHB with doses of 1, 10, and 30 µM were selected for the subsequent experiments.

#### 3.2.2 Effects of DPHB on glucose uptake in IR-3T3-L1 adipocytes

To explore the effect of DPHB on IR-3T3-L1 adipocytes, 1 μM Dex was used to induce IR in the 3T3-L1 adipocytes, and the intracellular glucose uptake was measured by the 2-NBDG method. In this experiment, the glucose uptake level of 3T3-L1 adipocytes was significantly decreased after 96 h of 1 μM Dex induction with or without insulin stimulation (*p* < 0.001) (Figure 4B), which implied that the IR model was successfully established. However, 1–30 μM DPHB was found to dose dependently reverse the Dex-treated decrease in the glucose uptake (*p* < 0.001). In addition, 30 μM of DPHB was found to increase the glucose uptake to the near-normal level (*p* < 0.001). Hence, the results indicated that DPHB could improve glucose metabolism and enhance insulin sensitivity of IR-3T3-L1 adipocytes induced by Dex.

#### 3.2.3 Effects of DPHB on glucose transporter type 4 in IR-3T3-L1 adipocytes

The insulin-responsive glucose transporter type 4 (GLUT4) plays a major role in glucose uptake and metabolism of the insulin target tissues, and the translocation of GLUT4 promotes glucose transport (Armoni et al., 2007; Lin et al., 2018). Therefore, to investigate the effect of DPHB on GLUT4 in IR-3T3-L1 adipocytes, the intracellular GLUT4 mRNA expression was determined by the qPCR method, and the protein expression of GLUT4 was measured by Western blotting. When compared with the control group, the GLUT4 mRNA expression was significantly decreased in the model group (*p* < 0.05), indicating the existence of IR. However, 1–30 μM DPHB was found to significantly reverse the Dex-treated GLUT4 mRNA expression, and 10 μM increased the GLUT4 mRNA expression by approximately seven-fold relative to that of the model group (*p* < 0.01) (Figure 4C). Interestingly, the results of Western blotting revealed that DPHB could significantly increase the protein expression of cytosol GLUT4 and membrane GLUT4 in IR-3T3-L1 adipocytes in a dose-dependent manner (*p* < 0.05). Overall, the results indicated that DPHB could effectively promote the translocation of GLUT4 to the cell membrane and thereby plays a role in promoting glucose uptake (Figures 4D–F).

#### 3.2.4 Effects of DPHB on lipid accumulation in IR-3T3-L1 adipocytes

**FIGURE 5:**
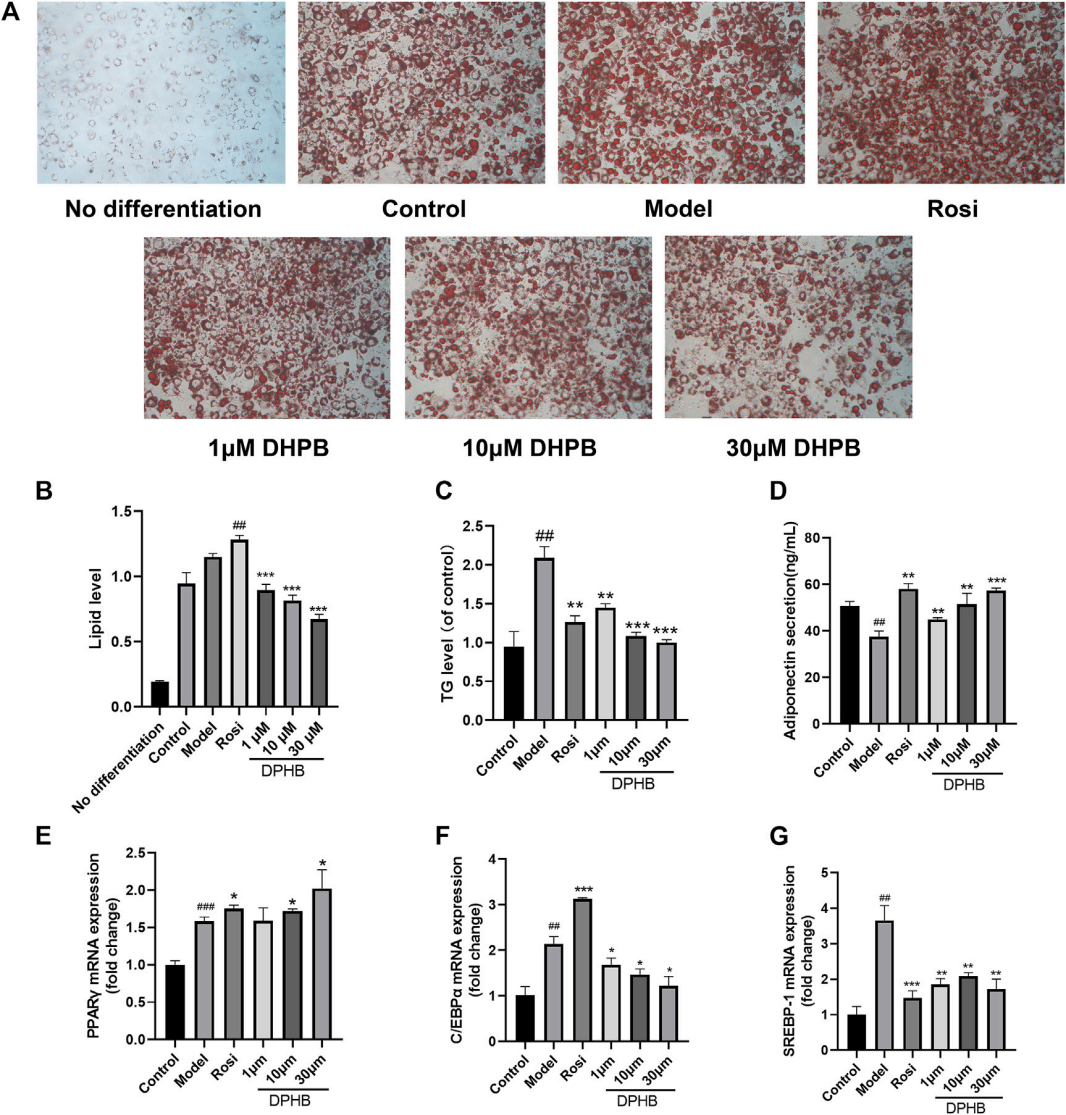
Effects of DPHB on lipid accumulation and adipokine expression in IR-3T3-L1 adipocytes. **(A)** Lipid accumulation was visualized by oil red O staining (magnification: ×100); **(B)** quantification of the lipid drop dissolved in isopropanol by detection at 510 nm; **(C)** accumulation of triglycerides; **(D)** adiponectin secretion; **(E–G)** mRNA expression of PPARγ, C/EBPα, and SREBP-1. All values are expressed as means ± SD, (*n* ≥ 3). *p < 0.05, **p < 0.01, and ***p < 0.001, when compared with the model control group; and ^##^ *p* < 0.01 and ^###^ *p* < 0.001, when compared with the control group.

Past network pharmacology discovered that the mechanism by which DPHB improves IR may be related to its involvement in cellular lipid responses, and that lipid metabolism disorder is one of the main causes of IR and obesity. Therefore, the effect of DPHB on lipid accumulation and TG content in IR-3T3-L1 adipocytes was investigated. Based on the oil red O staining results, the control group showed significantly more numbers of lipid droplets than the undifferentiated group, indicating that the cells were mature (Figure 5A). However, when compared with those of the control group, the lipid droplets in the model group were large and more in number, indicating that IR promoted lipid accumulation in 3T3-L1 adipocytes. In contrast, the size of lipid droplets of 3T3-L1 adipocytes in the 1–30 μM DPHB group was smaller, and its number was significantly less. In addition, the results of isopropanol quantification were found to be consistent with those of the oil red O staining (Figure 5B).

To quantify the intracellular lipid content, the TG levels were determined. We found that, when compared with that of the control group, the TG level of the model group was significantly increased (Figure 5C), implying that IR causes the accumulation of lipids in 3T3-L1 adipocytes. However, the 1–30 μM DPHB group showed significantly reduced TG content of IR-3T3-L1 adipocytes (*p* < 0.01), which is consistent with the results of oil red O staining. The aforementioned results indicate that DPHB improves lipid accumulation in IR-3T3-L1 adipocytes.

#### 3.2.5 Effects of DPHB on adipokines in IR-3T3-L1 adipocytes

Adiponectin, PPARγ, C/EBPα, and SREBP-1 are the key transcription factors of adipogenesis, which are all important adipokines involved in the homeostasis of glycolipid metabolism. To explore the effects of DPHB on lipid metabolism of IR-3T3-L1 adipocytes, the content of adiponectin secreted by the cells was measured by using the adiponectin ELISA kit, and the mRNA levels of PPARγ, C/EBPα, and SREBP-1 were quantified by the qPCR method. In the experiment, when compared with the control group, the model group showed significantly increased mRNA expression of *PPARγ*, *C/EBPα*, and *SREBP-1* and inhibition of the secretion of adiponectin (Figures 5D–G). However, DPHB could significantly promote the mRNA expression of *PPARγ* (*p* < 0.05) and adiponectin secretion (*p* < 0.01) as well as significantly inhibit the mRNA expression of *C/EBPα* (*p* < 0.05) and *SREBP-1* (*p* < 0.01). In addition, PPARγ and adiponectin could improve insulin sensitivity. Therefore, the aforementioned results indicated that DPHB could improve glucose and lipid metabolism disorders and improve insulin sensitivity by regulating some of the adipokines (such as adiponectin, PPARγ, C/EBPα, and SREBP-1).

#### 3.2.6 Effect of DPHB on the PI3K-AKT signaling pathway in IR-3T3-L1 adipocytes

**FIGURE 6:**
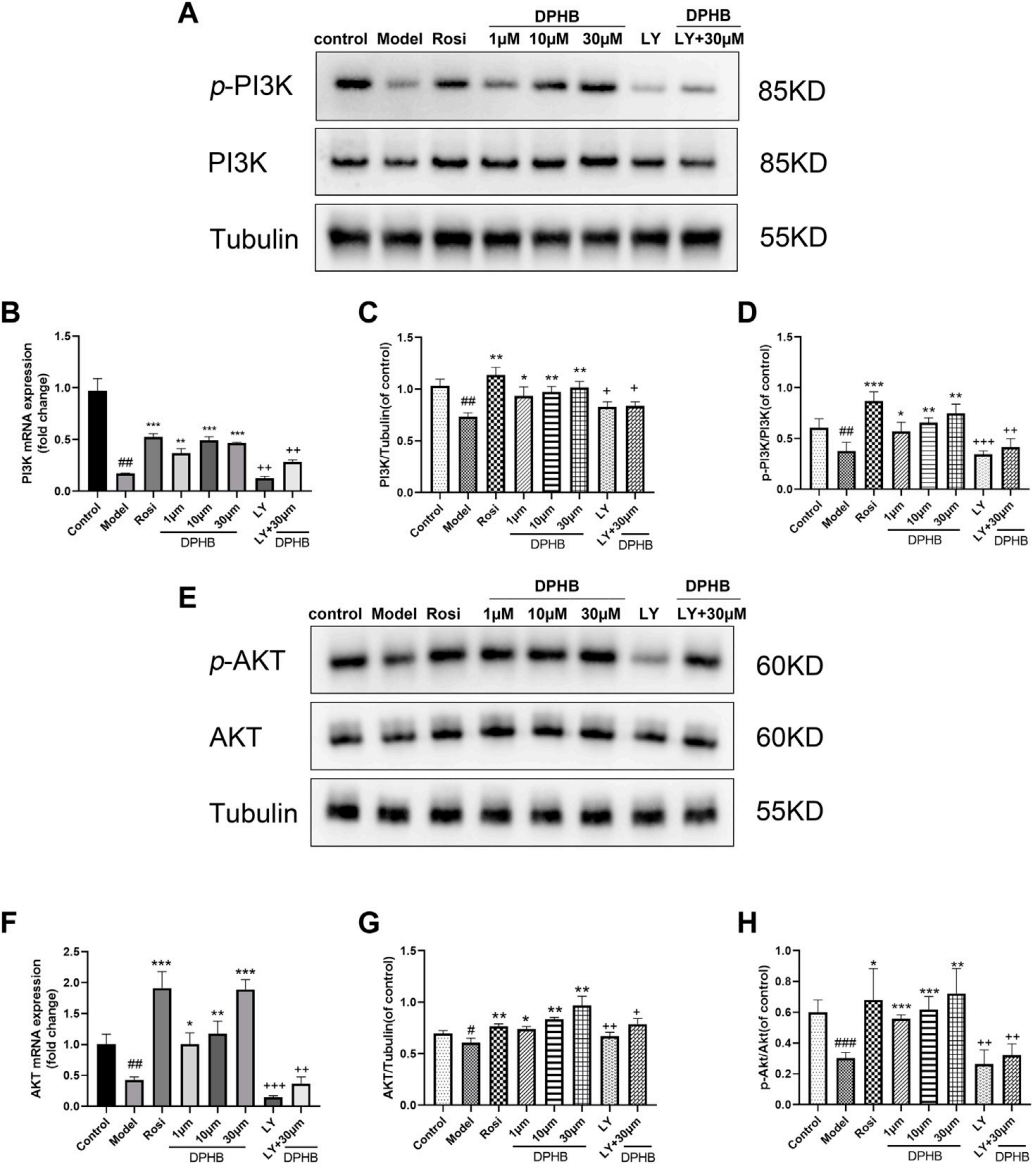
Effects of DPHB on the PI3K/AKT signaling pathway in IR-3T3-L1 adipocytes. **(A)** Protein expression of PI3K; **(B)** mRNA expression of PI3K; **(C)** quantification of PI3K; **(D)** quantification of *p*-PI3K; **(E)** protein expression of PI3K; **(F)** mRNA expression of AKT; **(G)** quantification of AKT; and **(H)** quantification of *p*-AKT. All values are expressed as means ± SD, (*n* ≥ 3). *p < 0.05, **p < 0.01, and ***p < 0.001, when compared with the model control group; ^##^ *p* < 0.01 and ^###^ *p* < 0.001, when compared with the control group; and ^+^ *p* < 0.05, ^++^ *p* < 0.01, and ^+++^ *p* < 0.001, when compared with the 30-μM DPHB group.

To further evaluate the molecular mechanisms of DPHB ameliorating IR, Western blotting was performed to examine the related targets of DPHB. The PI3K/AKT signaling pathway was identified by network pharmacology as a potential core pathway for DPHB toward improving IR. As shown in Figures 6A,C–E,G,H, when compared with the control group, the PI3K and AKT protein expressions were significantly decreased in the model group of Dex-treated 3T3-L1 adipocytes. On the other hand, after DPHB treatment, this decrease in the level was reversed, and the levels approached the basal level (*p* < 0.01). Moreover, DPHB could significantly increase the protein expressions of *p*-PI3K (Tyr458/Tyr199) and *p*-AKT (Ser473). Moreover, the mRNA expression of PI3K and AKT confirmed this result (Figures 6B,F).

To determine whether DPHB improves IR and lipid metabolism disorders depending on PI3K, the gene and protein expressions of PI3K were decreased by the PI3K inhibitor LY294002, followed by the observation of the effect of DPHB on the related proteins under the interference of PI3K inhibitors. LY294002 was found to significantly reduce the gene and protein expressions of PI3K (*p* < 0.01), indicating that LY294002 effectively inhibited the expression of PI3K. After DPHB treatment, although the expression of PI3K protein was only slightly increased, when compared with the non-inhibition group (30-μM DPHB group), the expression of PI3K protein was significantly decreased (*p* < 0.01), and no significant difference was noted relative to the LY294002 group. Moreover, AKT—the downstream target of PI3K—also showed this trend. Thus, our results indicated that DPHB plays a role in improving IR through the PI3K-mediated PI3K-AKT signaling pathway.

#### 3.2.7 Effect of DPHB on the TNF signaling pathway in IR-3T3-L1 adipocytes

**FIGURE 7:**
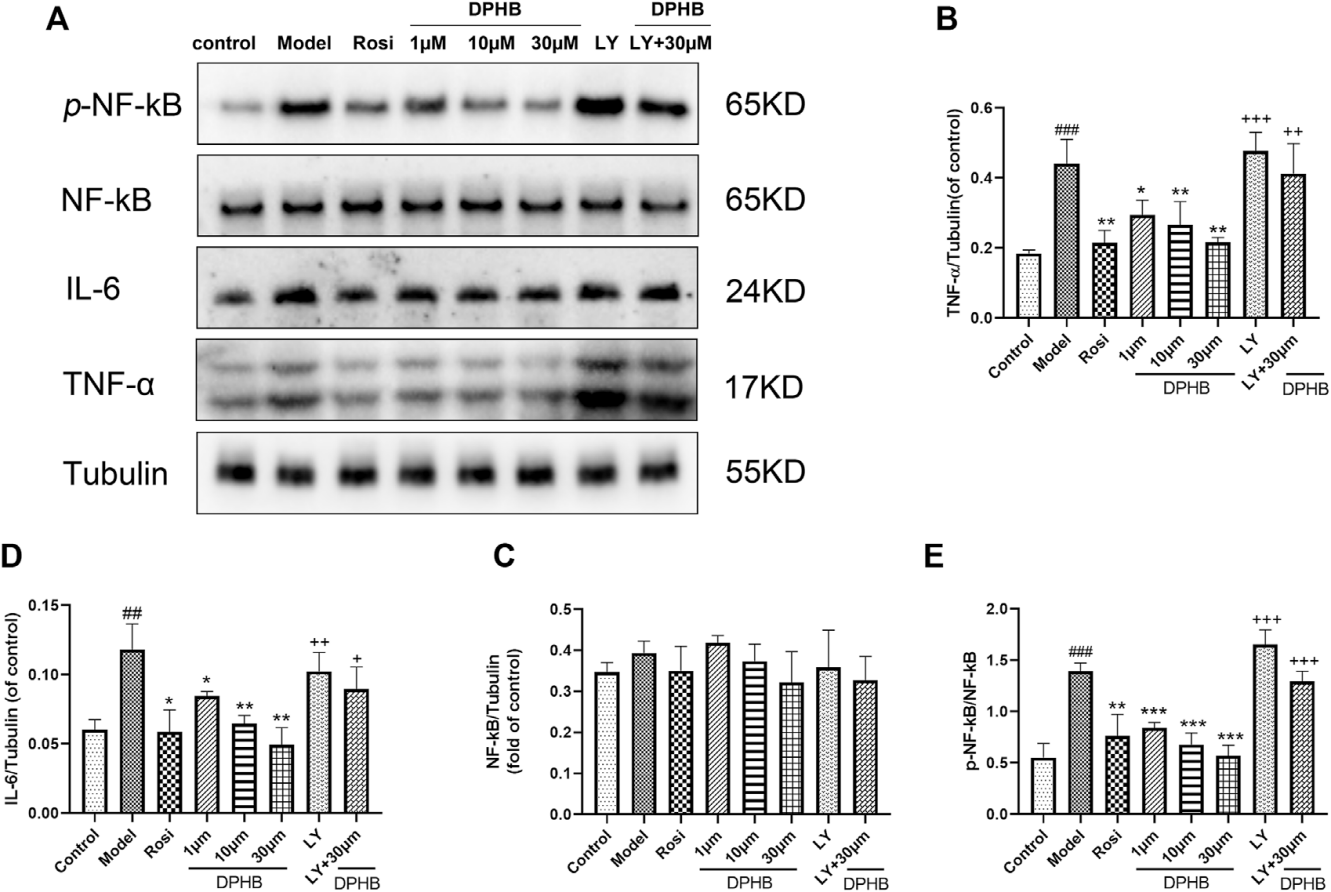
Effects of DPHB on the TNF signaling pathway in IR-3T3-L1 adipocytes. **(A)** Protein expression of TNF-ɑ, IL-6, NF-kB p65, and *p*-NF-kB p65; **(B)** quantification of TNF-ɑ; **(C)** quantification of IL-6; **(D)** quantification of NF-kB p65; and **(E)** quantification of *p*-NF-kB p65. All values are expressed as means ± SD, (*n* ≥ 3). *p < 0.05, **p < 0.01, and ***p < 0.001, when compared with the model control group; ^##^ *p* < 0.01 and ^###^ *p* < 0.001, when compared with the control group; and ^+^ *p* < 0.05, ^++^ *p* < 0.01, and ^+++^ *p* < 0.001, when compared with the 30-μM DPHB group.

In the aforementioned network pharmacology study, the TNF signaling pathway was a potential pathway for DPHB to improve IR. The phosphorylation of NF-kB P65 in the TNF signaling pathway promotes the secretion of inflammatory factors (such as TNF and IL-6) and reduces the body’s insulin sensitivity. Accordingly, we hypothesized that DPHB may improve IR by reducing the inflammatory response. Next, to verify this conjecture, Western blotting was performed to verify the key proteins of this pathway. When compared with the control group, the model group showed significantly increased protein expression of TNF-α, IL-6, and *p*-NF-kB p65 (Figure 7), indicating that the occurrence of IR aggravated the inflammatory response. Meanwhile, the protein expression of TNF-ɑ, IL-6, and *p*-NF-kB p65 was significantly inhibited after DPHB treatment. Furthermore, the total protein expression of NF-kB was not significantly affected by any treatment. These results confirm that DPHB could improve IR by alleviating inflammation.

In order to further investigate whether DPHB depends on PI3K to influence the TNF signaling pathway toward improving IR, the protein effects of DPHB on the related pathways were studied under PI3K inhibition. As shown in Figures 7A–E, under the inhibition of LY294002, the protein expressions of TNF-ɑ, IL-6, and *p*-NF-kB p65 were significantly increased, while treatment with 30-μM DPHB showed relative significantly decreased expression. However, no statistical difference was noted in the results relative to those of the LY294002 group. These results further indicated that the regulatory effect of DPHB on the TNF signaling pathway was dependent on the activation of PI3K, which demonstrates that impaired insulin signaling activates the TNF signaling pathway.

## 4 Discussion

The occurrence of IR leads to several metabolic disorders, such as hyperglycemia, hyperlipidemia, and hypertension (Pruimboom et al., 2017). IR is also considered to be the common pathological basis of many diseases such as T2DM, hypertension, coronary heart disease, metabolic syndrome, and polycystic ovary syndrome. Therefore, the study of pathogenesis and prevention of IR has great clinical significance. Obesity is not only recognized as a major factor in the development of IR but also as a chronic inflammatory state. White adipose tissue is an important endocrine organ producing proinflammatory cytokines and adipokines (Yang et al., 2019). When adipose tissue expands, adipocytes undergo hypertrophy and hyperplasia, and ectopic fat deposition occurs in the liver and skeletal muscle, leading to IR (Wijetunge et al., 2019). Moreover, cellular hypoxia and activation of local inflammation within adipose tissue occur when larger adipose cells exceed the local oxygen supply (Chan et al., 2019). The activation of the inflammatory response can reduce the body’s insulin sensitivity and abnormal glucose tolerance to varying degrees, thus aggravating IR (Petersen and Shulman, 2018). Therefore, limiting obesity and inflammation is considered an effective means to prevent the occurrence of IR.

Pharmacological PPI and C-T-P network analysis indicated that AKT1 and PI3KCA are the core targets of DPHB in IR. Impaired insulin signaling is the major cause of IR. The PI3K/AKT signaling pathway is an important pathway of insulin signaling, involved in glucose and lipid metabolism along with cell proliferation and survival (Huang et al., 2018). Under normal physiological conditions, insulin binds to insulin receptors on the membrane of target tissues, resulting in tyrosine phosphorylation of insulin receptor substrate 1(IRS-1), which activates PI3K and AKT, and promotes the transfer of GLUT4 from vesicles to the plasma membrane, facilitating glucose transport (Vlavcheski et al., 2020). Therefore, upregulation of PI3K-AKT signaling is considered one of the effective ways to treat IR. Interestingly, molecular docking studies showed that DPHB has a high binding affinity to PI3K and AKT. *In vitro* studies also showed that DPHB can activate PI3K and AKT phosphorylation in a dose-dependent manner, thus promoting glucose uptake and GLUT4 transport in IR-3T3-L1 adipocytes. Notably, the effect of DPHB on PI3K was reversed in the presence of the PI3K inhibitor (LY249002, 10 µM). These results suggest that DPHB helps in overcoming IR through the PI3K/AKT signaling pathway.

The GO analysis with a network pharmacology-based approach indicated that DPHB may play an anti-IR role by participating in the cell response to lipids, thus positively affecting the lipid metabolism under IR. Most of the energy is stored in fat tissue in the form of TGs, and the synthesis of lipids requires the involvement of adipokines, such as PPARγ, C/EBPα, SREBP-1, and adiponectin (Kojta et al., 2020). SREBP-1 affects adipogenesis by regulating the expression of adipogenic proteins and is positively associated with obesity (Rosen et al., 2000). C/EBPα adipokines are the major transcription factors/activators associated with adipogenesis in adipocytes (Jeon et al., 2004). PPARγ, a member of the nuclear receptor superfamily, selectively promotes adipogenesis in adipose tissue, thereby reducing circulating TGs and free fatty acids (Xing et al., 2015). In addition, PPARγ agonists can be used as insulin sensitizers to treat T2DM patients (Cheng et al., 2019). Adiponectin, an adipokine abundantly expressed in white and brown adipose tissue, has insulin-sensitizing and anti-inflammatory effects that can reduce TG levels and improve glucose and lipid metabolism (Li et al., 2020). Our results showed that DPHB reduces lipid accumulation, TG content, and the mRNA expression of *C/EBPα* and *SREBP-1* in IR-3T3-L1 adipocytes. It can also promote adiponectin secretion and enhance the mRNA expression of *PPARγ*. It further revealed that DPHB can exert anti-IR effects by improving the lipid response.

The KEGG enrichment analysis combined with network pharmacology indicated that TNF signaling was one of the important pathways in the role of DPHB in improving IR. The tumor necrosis factor (TNF) superfamily is closely related to inflammation and is involved in a range of inflammatory and immune responses (Holbrook et al., 2019). When the cells received external stimulation with inflammatory factors (such as TNF-α and IL-6), NF-kB P65 is phosphorylated and translocated to the nucleus, thereby upregulating the expression of TNF-α and IL-6 as well as exacerbating the inflammatory response (Sun, 2017). The inhibition of TNF-α could upregulate AKT phosphorylation in IR-HepG2 cells and improve lipid-induced insulin resistance (Hotamisligil et al., 1996). IL-6 inhibits the insulin signaling cascade and inhibits the phosphorylation of AKT, ultimately deepening the development of IR (Senn et al., 2003). Consistent with the aforementioned predictions, the molecular docking results indicated that DPHB had better binding ability toward TNF, IL-6, and RELA. Interestingly, our results indicated that DPHB can significantly inhibit the protein expression of TNF-α, IL-6, and *p*-NF-kB p65. It further suggested that the anti-IR effect of DPHB is exerted by alleviating the inflammatory response. Investigation with PI3K inhibitors revealed that TNF signaling is also regulated by PI3K; therefore, inhibition of PI3K promotes the development of IR and aggravates the inflammatory response. This also further showed a tight link between IR and inflammation.

This study has some limitations. The public databases employed in this study are real-time updates, and the target data of network pharmacology may change in the future. Moreover, only molecular docking and *in vitro* experimental validation were performed, and subsequent validation should be combined with animal models. We will thereby aim to pursue these gaps as a focus of our future work to provide more reliable data supporting the elucidation of the treatment of IR with DPHB.

## 5 Conclusion

**FIGURE 8:**
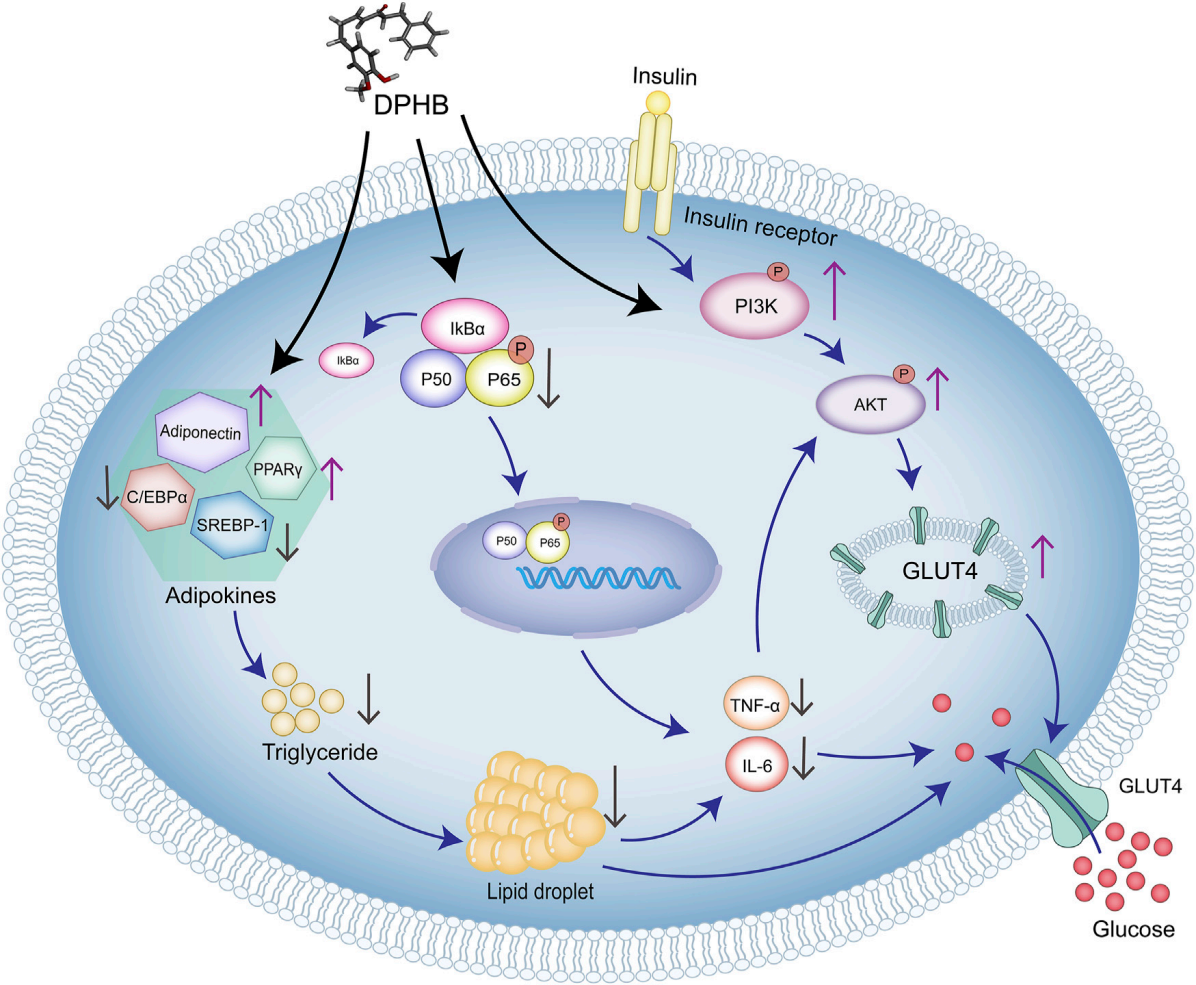
Molecular mechanism of DPHB alleviating insulin resistance through PI3K/AKT and TNF signaling pathways.

In this study, network pharmacology and molecular docking were used to predict the molecular mechanism of DPHB in alleviating IR. *In vitro* analysis confirmed that DPHB could improve insulin resistance and glycolipid metabolism disorders through PI3K/AKT and TNF-α signaling pathways. Furthermore, the interference of PI3K inhibitors further confirmed that this activity of DPHB was dependent on the activation of PI3K (Figure 8). In conclusion, this study revealed the effect of DPHB on IR and provided a new reference for the anti-IR biological activity of DPHB, along with a theoretical basis for future experimental research and clinical applications of DPHB.

## Data Availability

The original contributions presented in the study are included in the article/Supplementary Material; further inquiries can be directed to the corresponding authors.
